# Modelling the mass consumption potential of organic food: Evidence from an emerging economy

**DOI:** 10.1371/journal.pone.0291089

**Published:** 2023-09-01

**Authors:** Qing Yang, Lyu Xinyue, Mohammad Enamul Hoque, Abdullah Al Mamun, Muhammad Khalilur Rahman, Jian Yao

**Affiliations:** 1 UKM—Graduate School of Business, Universiti Kebangsaan Malaysia, UKM Bangi, Selangor Darul Ehsan, Malaysia; 2 BRAC Business School, BRAC University, Dhaka, Bangladesh; 3 Faculty of Entrepreneurship and Business, Universiti Malaysia Kelantan, Pengkalan Chepa, Malaysia; 4 Angkasa-Umk Research Academy (AURA), Universiti Malaysia Kelantan, Pengkalan Chepa, Malaysia; St John’s University, UNITED STATES

## Abstract

The deterioration of the environment, shortage of resources, and frequent occurrence of food safety issues have made people increasingly concerned about themselves while maintaining their health and protecting the environment through food. Organic food, as a healthy and eco-friendly option, is gradually gaining attention. Based on the value-belief-norm theory, this study explores why individuals consume organic food and the range of factors that lead to this consumer behavior. This study adopted a cross-sectional design and collected quantitative data from conveniently selected 300 youth participants in Bangladesh using an online survey. The findings revealed that health values and motivation have a significant positive effect on healthy eating beliefs, which, together with the awareness of the consequences, affect personal norms toward organic food consumption. Personal norms also have a significant positive effect on organic food consumption behavior among Bangladeshi youth. Finally, trust on organic food positively moderates the effect of personal norms on organic food consumption. The findings of this study are expected to foster the development of a comprehensive framework to promote programs and policies focused on organic and healthy food consumption culture among youth in developing nations.

## Introduction

Since the 21st century, human societies have experienced rapid growth in productivity, population expansion, and overconsumption of resources, leading to a gradual deterioration in environmental quality and repeated food safety incidents, which have seriously affected the sustainable development of the world economy and people’s health [1, 2]. It can be argued that these situations, which threaten human health, result from people’s non-consideration of the long-term ill-effects of irresponsible production and consumption [3]. To address these challenges, people are focusing on waste disposal methods that improve the environment and benefit health [4]. Food, as a basic need in people’s daily lives, is also a main focus of the scholars exploring sustainability and health [5].

Many researchers have been at length to point out that economic growth in the vast majority of developing countries has been accompanied by severe environmental degradation due to a lack of good foresight [6–8]. After a stable economic development, a special fund has to be earmarked for the improvement and restoration of the damaged environment [9]. Meanwhile, owing to personal health and ecological sustainability concerns, there has been a widespread increase in the demand for organic food worldwide, with an increasing number of consumers becoming aware of its benefits [10, 11]. In some developing Asian countries, organic food has received much attention from consumers, and the organic food market has grown rapidly [12]. There have been many attempts to explore and explain the motivations behind consumers’ purchasing behavior; however, most studies have focused on developed countries, which may be related to their enormous demand [13]. The consumption of organic food is still a new practice in many developing countries [14]. This has resulted in less information and research on consumer behavior toward organic food in developing countries (e.g., Bangladesh). Moreover, countries’ cultures can influence consumers’ green consumption behavior [15]. The market economy is a demand-oriented economy, and consumer demand is the starting point of all market orientation [16]. In this context, it is particularly important to study the reasons that influence Bangladeshi consumers to consume organic food.

The norm activation model (NAM) and value-belief-norm (VBN) theory have often been used in studies relating to pro-environmental and healthy eating behaviors and intentions [17, 18]. Ascription of responsibility (AR) is often used in much of the literature as a mediating variable between awareness of consequences (AC) and personal norms (PN) [19]. In addition, when exploring people’s choice of organic food, scholars tend to explore the influence of pro-environmental altruistic values on consumption behavior [20]. Based on these gaps in the extant literature, this study explores the factors that influence the organic food consumption by the youth in Bangladesh. This study tests the VBN theory while highlighting the importance of health values (HV) and health motivation (HM) on healthy eating beliefs (HE), and hopes to test whether awareness of consequences and ascription of responsibility can independently influence personal norms in our survey sample. In addition to these factors, green trust is an important variable that influences people’s choice to purchase environmentally friendly products [21, 22]. This study argues that trust on organic food (GT) may play a moderating role between personal norms and consumption of organic food, and it attempts to demonstrate the existence of this relationship.

The remainder of this study is structured as follows. Section 2 describes the two theoretical foundations of this study, namely the VBN theory and NAM, and the research hypotheses. Section 3 discusses the collected data and the methods used to test the hypotheses. Section 4 reports the analyses results. Section 5 discusses the results, explores the limitations of this study, and offers possible directions for future research.

## Literature review

### Theoretical foundation

The basis of the VBN theory was developed from the norm activation model proposed by Schwartz [23]. NAM was initially developed to explain how altruistic intentions and behaviors are formed in a pro-social context. It consists of three main concepts: awareness of consequences, ascription of responsibility, and personal norms [23]. Personal norms are the expectations of individuals to perform a particular behavior in a given situation [24]. When not acting prosocially, awareness of consequences is defined as whether an individual is aware of the negative consequences for others [18]. Ascription of responsibility is described as an individual’s sense of responsibility for the negative consequences of failing to perform a specific target behavior [25]. Previous research has determined that normative activation theory (NAM) is applicable to predict individuals’ pro-environmental and pro-social behaviors which are significantly influenced and moderated by their beliefs and personal norms, such as awareness of consequences and ascription of responsibility. This has been confirmed in recent years by studies such as urban residents’ behavior when purchasing energy-efficient appliances [26], pro-environmental intentions and behaviors in the public sphere [27], and consumers’ intentions and behaviors when choosing organic food restaurants [28].

VBN theory is a causal chain theoretical framework proposed by Stern et al. [29] based on the Normative Activation Model (NAM) that links individuals’ values, belief structures, and norms that support and uphold the environment/society to their pro-environmental/social behaviors, which in turn explains the outcomes of human-environmental and social interactions. The VBN theory has been applied to study pro-environmental behavior and sustainable consumption, including improving household energy efficiency [30], policies to reduce car use [31], and the purchase of sustainable masks [32] and organic food [33]. Considering that organic food consumption is a specific type of pro-environmental behavior and that organic food production reduces dependence on chemical fertilizers, pesticides, and genetically modified crops and reduces pollution of soil and water sources [34], the VBN model was used in this study. This study extends the NAM and VBN models by adding health values, health motivations, and healthy eating beliefs, aiming to more fully explain how consumers’ health values, health motivations, and healthy eating beliefs interact with personal norms, perceptions of consequences, and attributions of responsibility and influence subsequent consumption intentions and behaviors toward healthy foods (organic foods). Among these, health values refer to the importance or priority consumers assign to their health and well-being relative to other aspects of their lives [35]; health motivation refers to the intrinsic or extrinsic factors that drive consumers to pursue health goals and overcome barriers [36]; and healthy eating beliefs refer to the factors that influence how consumers health benefits, risk perceptions, and emotional factors that influence how consumers view and perceive healthy foods [37].

In addition to the VBN theory, the Health Belief Model (HBM) adequately explains the relationship between health beliefs and personal norms. The health belief model suggests that individuals’ health behaviors are influenced by their subjective assessments of disease susceptibility, severity, benefits, and barriers [38]. The health belief model suggests that these beliefs influence individuals to adopt preventive or promotional behaviors, such as purchasing and consuming healthy foods. An important factor is personal norms, which refer to the extent to which individuals feel they should or should not adopt certain behaviors [38]. By combining the health belief model and the VBN model, Kim et al. [39] found that the inclusion of values and health beliefs can more effectively explain and promote people’s subsequent health behaviors if they are consistent with consumers’ values, beliefs, norms, attitudes, and motivations and take into account their perceived behavioral control and trust in health information or products. Therefore, this study combines the Health Belief Model (HBM) and Normative Activation Theory (NAM) based on VBN theory, and thus more deeply explores the relationship between health values, healthy eating beliefs, awareness of consequences, ascription of responsibility, and personal norms among Bangladeshi youth based on the VBN theory.

### Hypotheses development

#### Health values (HV) and healthy eating belief (HEB)

Health values can be described as an individual’s perception of taking healthy actions to prevent disease and maintain health, and the degree to which health is valued [40]. Health values generally reflect individuals’ perceptions of the importance and priority of health and the value or usefulness of healthy behaviors [41]. Health values can motivate individuals to pursue their health goals and facilitate their alignment with their physical and mental states [42]. Health values can also influence individuals’ beliefs about health issues and behaviors, i.e., their cognitive and affective factors about a health issue or behavior, such as the benefits of disease management and prevention, healthy weight maintenance, and healthy eating to increase life expectancy [41]. Healthy eating beliefs in this study refer to the individuals’ unwavering mindset that purchasing and consuming healthy foods is helpful for health management.

In VBN theory, a person’s values strongly influence her beliefs [29]. For example, Carfora et al. [42] demonstrated that an individual with biosphere and altruistic values is more likely to have beliefs that support environmental protection and animal harm reduction. Similarly, Chen [43] showed that people’s biosphere and altruistic values may contribute to their belief in their personal responsibility to reduce threats that affect the environment. These studies suggest that there is a strong link between values and beliefs and that this link may be generalized across domains and topics. This study is concerned with the relationship between values and beliefs in the health domain, particularly those related to healthy eating. People who value a nutritious diet and understand the impact of nutrition on health are more likely to eat healthily and actively engage in healthy behaviors [44]. Organic food consumers value knowing where their food comes from and their relationship with farmers, and they believe that buying organic is better for their health and the environment. These values that emphasize maintaining health and environmental friendliness support their belief in buying organic food [33]. Therefore, this study can hypothesize that individuals with health values will have stronger beliefs about healthy eating. Based on the above, the following hypothesis was proposed.

**H**_**1**_: *Health values positively influence healthy eating belief*.

#### Health motivation (HM) and healthy eating belief (HEB)

Health motivation refers to the goal orientation of consumers’ actions toward health protection [45]. Health motivation refers to the degree of concern and intensity of consumers’ pursuit of their health and well-being, as well as intrinsic or extrinsic drivers of health behavior [46]. Health motivation can motivate consumers to acquire and process health-related information, increase their knowledge and understanding of health and healthy eating, and promote the development of more stable and enduring attitudes and behaviors [47]. Health motivation can also be influenced by personal characteristics and environmental factors, such as gender, age, education level, cultural background, and social support [48]. In the present study, the relationship between health motivation and healthy eating beliefs was focused on and explored, especially beliefs related to the purchase and consumption of healthy foods. This study hypothesized that health motivation can influence consumers’ perceptions and affective factors about healthy foods, such as their assessment of their nutritional value, safety, taste, and price. According to the Health Belief Model (HBM), health motivation enables individuals to hold reasonable beliefs about health issues, such as susceptibility to disease, severity, benefits, and barriers, which in turn influence individuals to adopt preventive or promotional behaviors, such as purchasing and consuming healthy foods [49]. Based on the above review, we hypothesized that consumers with strong health motivation would have more positive beliefs about healthy eating. Therefore, we propose the following hypothesis.

**H**_**2**_: *Health motivation positively influences healthy eating belief*.

#### Healthy eating belief (HEB) and awareness of consequences (AC)

Healthy eating beliefs usually refer to an individual’s unwavering belief that purchasing and consuming healthy foods is helpful for health management, and reflect the individual’s perceptions and emotional factors about healthy foods, such as assessment of their nutritional value, safety, taste, and price [47]. Healthy eating beliefs can influence consumers’ attitudes toward healthy eating and can also be influenced by personal needs and environmental factors such as gender, age, education level, cultural background, and social support [48]. This study focused on the relationship between healthy eating beliefs and consequence awareness, especially in relation to unhealthy eating. Awareness of consequences refers to the extent to which individuals are aware of the negative health outcomes of their unhealthy behaviors (especially diet). Consequence awareness reflects an individual’s awareness of the adverse outcomes of an event for others or for nonhumans (e.g., the environment) [29]. It also can motivate individuals to develop personal norms and obligations to act in ways that lead them to adopt behaviors that are beneficial to the environment and health [42]. Youn et al. [50] studied consumers’ willingness to consume in traditional restaurants and defined the awareness of consequences as an individual’s perception of the negative results caused by others or the environment. Considering the interpretation of previous literature and the need for further research, this study defines awareness of consequences as the extent to which individuals are informed of the negative health outcomes of their unhealthy behaviors (especially their diet).

According to the VBN theory, there is a significant correlation between individuals’ beliefs and their awareness of consequences [29]. For instance, in case of individuals choosing to consume meat, the stronger their belief in reducing meat consumption, the more likely they are to be motivated by moral obligations and to think about the possible negative environmental impacts of their consumption behavior [51]. Consumers with health beliefs also think about the negative effects of unhealthy diets on their overall health [52]. Furthermore, egoists are more concerned about their dietary health than altruists are, and they are more likely to choose organic food owing to the health problems associated with an unhealthy diet [53]. Therefore, this study argues that individuals who believe in healthy eating are more likely to have a clear understanding of the negative consequences of unhealthy eating and proposes the following hypothesis.

**H**_**3**_: *Healthy eating belief positively influences awareness of consequences*.

#### Awareness of consequences (AC) and ascription of responsibility (AR)

Health benefits have become a reference criterion for food choices [54]. Organic food, which theoretically improves both the environmental impact and nutritional outcomes, is more popular among consumers who are aware of health consequences [55]. People consider the consequences of consuming food for their own and their families’ health based on their health concerns, thus stimulating their interest or actions to seek information and knowledge about organic food [56]. Evidently, people’s consumption of organic food is mainly related to their self-interest motivation, and their concern about the relationship between organic food and personal health issues [57]. This is similar to the attribution of personal responsibility for one’s own health. Studies have pointed out that the greater an individual’s ability to perceive problems, the more inclined they are to take responsibility [58]. The causal relationship between the awareness of consequences and ascription of responsibility has also been mentioned in both the normative activation theory and belief-value-normative theory [23, 29]. Therefore, this study proposes that individuals’ concerns about adverse health consequences can influence their attribution of health responsibility. Therefore, we propose the following hypothesis.

**H**_**4**_: *Awareness of consequences positively influences ascription of responsibility*.

#### Healthy eating belief (HEB) and personal norms (PN)

Organic foods are often considered safer than the conventional alternatives and offer greater health benefits [59]. Consumers are always willing to pay more for products that they perceive as healthy [60]. When individuals are aware of the benefits of organic food, their healthy eating belief drive them to develop a positive attitude toward consuming organic food owing to their self-interest, and thus, the consumption of organic food occurs later [61]. This suggests that individuals’ beliefs about healthy eating can motivate their desire to protect their health and that of their families. It has also been suggested that consumers who aim for a healthy diet may also perceive healthy foods as being associated with a healthy life, and thus expect to increase their life satisfaction in terms of health through the consumption of healthy foods [62]. According to VBN theory, individuals’ beliefs activate their personal norms [29], and further this study predicts some associations between healthy eating beliefs and personal norms, especially those related to the purchase and consumption of healthy foods. Yazdanpanah et al. [49] used the health belief model to confirm that beliefs about disease susceptibility and severity beliefs, as well as beliefs about the benefits and barriers to healthy eating, were significantly and positively associated with personal norms. Although the relationship between healthy eating beliefs and personal norms could not be discerned directly in the extant literature, this study combines the VBN theory and the Health Belief Model, to understand the relationship between healthy eating beliefs and personal norms, and then hypothesizes that individuals who believe in healthy eating are more likely to develop personal norms that are consistent with. As a result, the following hypothesis is proposed.

**H**_**5**_: *Healthy eating belief positively influences personal norms*.

#### Awareness of consequences (AC), ascription of responsibility (AR), and personal norms (PN)

It is an accepted view that personal norms are activated when individuals become aware of the negative consequences of their actions as well as believe that they should take responsibility for the consequences and that their actions can play a role in reducing these problems [31]. That is, personal norms are formed after internalizing the information that makes the individual aware of the outcomes of their behavior and the responsibility that comes with performing it, thus motivating behavior through guilt and shame [63]. Personal norms are more robust when individuals realize that they can contribute to reducing the problems [64]. The relationship between awareness of consequences, ascription of responsibility, and individual norms has been supported by a large body of literature on the norm activation model and VBN theory [34, 65]. In terms of healthy behavior, individuals have a moral responsibility to maintain a healthy lifestyle, especially when making food choices that account for the consequences of a non-healthy diet, which also demonstrates their personal norms [66]. For instance, Shin et al. [28] suggest that individuals’ awareness of the consequences of consuming non-organic food and the responsibility to cope with them, motivate their personal norms when choosing items from a menu, which ultimately leads to the choice of organic food. Therefore, this study argues that both awareness of consequences and ascription of responsibility are predictors of personal norms. As a result, the following hypotheses are proposed.

**H**_**6**_: *Awareness of consequences positively influences personal norms*.
**H**_**7**_: *Ascription of responsibility positively influences personal norms*.

#### Personal norms (PN) and organic food consumption (OFC)

The VBN theory suggests that personal norms directly predict the behavior [23]. Individuals develop anticipated feelings of guilt or pride about their actions before engaging in a behavior, and these anticipated emotions lead to the actual behavior [28]. For example, individuals who anticipate recognizing health risks associated with not exercising are likely to engage in exercising [63]. A similar situation exists with regard to the consumption of organic food. Individuals’ reflection on the impact of their food choices on themselves and on the environment may lead to a preference for organic food [67]. Thøgersen and Ölander [68] suggest that the stronger the personal norms of the consumers in favor of organic food, the more likely they are to increase their consumption of organic food and abandon their conventional food habits. Several studies have empirically examined the relationship between personal norms and consumers’ organic food consumption behavior using VBN theory, the TPB model, or a combination of both, and found that consumers’ personal norms have a significant positive impact on their intention to purchase organic food. In particular, consumers’ personal norms can motivate and promote subsequent consumption behaviors by prompting moral obligations towards behaviors such as eating healthy food, and creating a strong intention to consume organic food [20, 34]. Therefore, this study argues that the stronger the personal norms, the more likely is organic food consumption. Therefore, the following hypothesis is proposed.

**H**_**8**_: *Personal norms positively influence organic food consumption*.

#### The moderating effect of trust on organic food (GT)

Combined with Hart and Saunders’s [69] definition of trust, trust on organic food can be interpreted as an individual’s willingness, desire, and ability to rely on organic food and to accept the lowest level of vulnerability of organic food based on quality, reliability, and health features. Consumers’ trust in organic quality is a key factor influencing their decision to purchase organic products; the higher the level of trust, the higher the perceived monetary value of organic food for individuals [70]. This is because most consumers cannot verify the context in which food is produced, and they need to believe that the food they are buying is authentic before they can make up their minds to consume it [34]. Trust, as an intrinsic motivation for consumers, can facilitate the transition from the idea of buying organic food to consumption behavior [71]. Klöckner and Ohms [72] find that although personal norms can positively influence consumers to purchase organic milk, a lack of trust on organic milk can weaken the influence of personal norms on consumption. Based on the aforementioned literature, the following hypothesis is proposed.

**H**_**9**_: Trust on organic food moderates the relationship between personal norms and organic food consumption.

**Fig 1:**
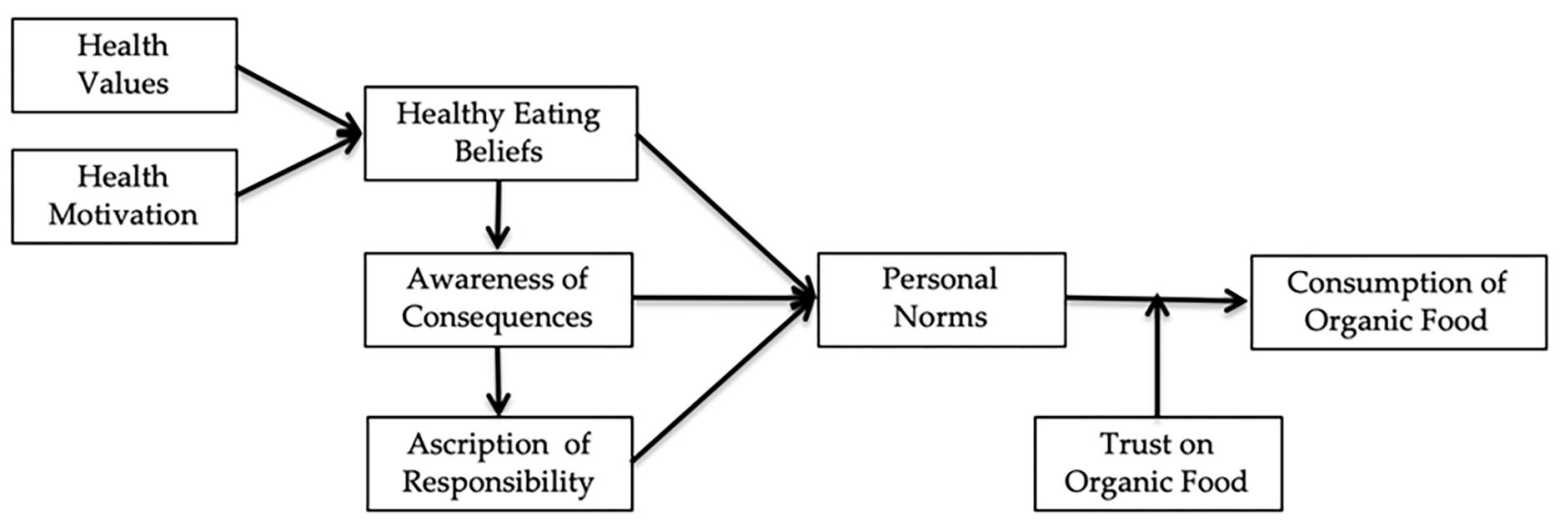
Research framework.

All associations hypothesized, are presented in Fig 1 below.

## Method

### Sample selection and data collection

The younger generation is increasingly embracing organic food consumption. Younger individuals are more likely to be health-conscious and environmentally aware, leading to a greater preference for organic and sustainable food options [73, 74]. Therefore, we select youth population in Bangladesh as our unit of analysis. According to the Ministry of Youth and Sports in Bangladesh, the age of youth is between 18–35 years [75]. A quantitative research method was used to investigate the influence of different VBN factors on consumers’ dietary habits regarding organic food consumption. The main data collection tool used in this study was a meticulously designed and meticulously executed structured questionnaire. There were two primary aspects to the questionnaire. Section 1 had statements that represented the variables studied in the investigation. Section 2 included questions on the respondents’ demographic information.

Consider the availability of target population and cost efficiency of the data collection process, this study conducted the convenience sampling technique which belongs to the non-probability sampling method. As a sampling method that emphasized in generalizability, convenience sampling technique meet the requirement of this quantitative study [76]. Therefore, the online survey link that generated from Google Forms were posed on some popular social media platform such as Facebook or Instagram, and voluntary participation by potential respondents. Upon accessing the questionnaire link, participants are required to provide their informed consent by signing a form prior to proceeding to the formal stage of questionnaire completion. Furthermore, a preliminary screening question was employed to ascertain the inclusion of only participants aged 18 and above in the study. No rewards, whether monetary or non-monetary, were provided as an incentive for participating in the study. A statement explicitly indicated that the data was being collected solely for academic purposes. Participants were guaranteed confidentiality and anonymity throughout the study. Participants have the option to withdraw from the study at any point during the period of the research.

Following the guidance and recommendations of Faul et al. [77] for G*Power, this study used the software to calculate the minimum sample size required for data analysis. With an effect size of *f*^*2*^ = 0.15, α = 0.05, and Power value = 0.80, the minimum sample size was calculated to be 103, in conjunction with the seven predictors of the study. Given the potential enhancement of statistical power associated with an increased sample size in convenient sampling [76], this study acquired 300 valid questionnaires subsequent to data screening procedures, which involved identifying and excluding instances of hasty responses or same way in item responses. The data for this study were collected from March to August 2022 through online survey. The human research ethics committee of Changzhi University approved this study (approval number: CZ-2022-0076).

### Survey instrument

This study has been adopted from other scholars’ scales to achieve its validity and reliability. Health values were measured using the scale designed by Lau et al. [40], and health motivation was measured using the scale given by Choi et al. [78]. Healthy eating beliefs were derived from the questionnaire scale of Han et al. [17]. The measurements for the awareness of consequences and ascription of responsibility were adapted from Stern et al. [29]. The items for personal norms were based on the scales of Choi et al. [79] and Ünal et al. [31]. The scale for trust on organic food was adapted from the Green Trust Scale designed by Chen [80]. These items (S1 Table) are measured using a five-point Likert scale.

A comprehensive three-stage pretesting approach was carried out to confirm the survey instrument’s face validity and content validity. The survey instrument was meticulously evaluated in the first phase by a panel of scholarly academics specialized in organic food consumption, consumer behavior, and research method. In the second step, two organic food traders were asked to carefully read the survey instrument to ensure that the content and language structure were clear. The subsequent phase of pre-testing involved the administration of the questionnaire to a cohort consisting of 30 individuals who are actively involved in the pursuit of a master’s degree. Finally feedback on survey instruments and measurement items effectively integrated.

### Multivariate normality

This study used the online Web Power tool to verify the multivariate normality of the data collected, although there are no criteria for such analysis in the PLS. The multivariate normality test results show that the p-values for Mardia’s multivariate skewness and kurtosis were < 0.05, thus indicating non-normality [81].

### Data analysis method

An increasing number of social science studies have used partial least squares structural equation modelling (PLS-SEM). PLS-SEM is based on the variance of the data, and it evaluates the model parameters by interpreting and using total variance. It is suitable for exploring data that lack the properties of normal distribution and that involve complex structural models containing many structures, indicators, and relationships; it is valid for both small datasets and large sample sizes [82]. This study had multivariate non-normality and structural complexity; thus, we chose PLS-SEM to test this research model.

## Results

### Demographic characteristics

The respondents’ demographic characteristics are presented in Table 1. The sample consisted of 108 men (36.0%) and 192 women (64.0%). There were slightly more female respondents than male respondents. With regards to age, 100 (33.3%) respondents were aged 18–25 years, 119 (39.7%) were aged 26–20 years, and 81 (27.0%) were aged 31–25 years. Among them, 117(39.0%) respondents had a household income below BDT10,000; 62 (20.7%) between BDT10,001 and BDT20,000; 54 (18.0%) between BDT20,001 and BDT30,000; 48 (16.0%) between BDT30,001 and BDT40,000, and 19 (16.0%) between BDT40,000 and BDT40,000. The number of students with a household income above BDT40000 was 19 (6.3%). Their educational level are as follows: secondary school certificate (15.0%), higher secondary certificate (6.0%), bachelor’s degree or equivalent (34.3%), master’s degree (41.7%), and doctoral degree (3.0%). The majority of the respondents were students (42.7%), private sector employees (29.0%), and public sector employees (22.3%). In addition, 18 (6.0%) of the respondents were self-employed.

**Table 1:** Demographic characteristics.

	N	%		N	%
*Gender*			*Education*		
Female	108	36.0	Secondary School Certificate	45	15.0
Male	192	64.0	Higher Secondary Certificate	18	6.0
Total	300	100.0	Bachelor’s Degree or equivalent	103	34.3
			Master’s Degree or equivalent	125	41.7
*Age Group*			Doctoral Degree or equivalent	9	3.0
18–25 Years	100	33.3	Total	300	100.0
26–30 Years	119	39.7			
31–35 Years	81	27.0	*Occupations*		
Total	300	100.0	Student	128	42.7
			Private Service	87	29.0
*Income*			Public Service	67	22.3
Below BDT10000	117	39.0	Self Employed	18	6.0
BDT10001 to BDT20000	62	20.7	Total	300	100.0
BDT20001 to BDT30000	54	18.0			
BDT30001 to BDT40000	48	16.0			
More than BRT40000	19	6.3			
Total	300	100.0			

**Note:** 1 USD = 102 BDT

### Validity and reliability

Table 2 presents the results of the tests for reliability and convergent validity of the questionnaire. First, Cronbach’s alpha, Dijkstra–Hensele’s *rho*, and composite reliability for each variable were all greater than 0.7, indicating the reliability of the questionnaire’s internal consistency. Second, the mean variance extracted for each latent variable was greater than 0.5, suggesting good convergent validity.

**Table 2:** Reliability and validity.

Variables	No. Items	Cronbach’s Alpha	Dijkstra-Hensele’s *rho*	Composite Reliability	Average Variance Extracted
**Health Values**	4	0.959	0.961	0.970	0.891
**Health Motivation**	5	0.884	0.923	0.909	0.667
**Healthy Eating Belief**	5	0.915	0.941	0.942	0.770
**Awareness of Consequences**	5	0.965	0.965	0.973	0.876
**Ascription of Responsibility**	5	0.949	0.953	0.961	0.831
**Personal Norms**	5	0.961	0.961	0.970	0.864
**Trust on Organic Food**	5	0.961	0.962	0.970	0.865
**Organic Food Consumption**	5	0.955	0.955	0.965	0.847

This study also examined the discriminant validity of the scales, as shown in Table 3. As this study uses a reflexive measurement model, the Fornell-Lacker criterion was first used to measure discriminant validity. The values of each variable on the diagonal of Table 3 are greater than the correlation coefficients of other variables in each column. Thus, the discriminant validity for each variable is considered good. Heterogeneous single trait ratio (HTMT) is used to measure discriminant validity in this study. The results show that the HTMT between all constructs is less than 0.9; therefore, it is concluded that the discriminant validity is also excellent. The cross-loading of the indicators (S2 Table) also confirms the good discriminant validity of the questionnaire items.

**Table 3:** Discriminant validity.

Variables	HV	HM	HE	AC	AR	PN	GT	OFC
**Fornell-Larcker Criterion**								
**HV**	0.944							
**HM**	0.802	0.817						
**HE**	0.776	0.718	0.877					
**AC**	0.813	0.751	0.827	0.936				
**AR**	0.667	0.659	0.636	0.738	0.912			
**PN**	0.707	0.698	0.765	0.720	0.579	0.930		
**GT**	0.673	0.642	0.759	0.728	0.562	0.790	0.930	
**OFC**	0.579	0.571	0.660	0.621	0.500	0.754	0.761	0.920
**Heterotrait-Monotrait Ratio (HTMT)**								
**HV**	-							
**HM**	0.815	-						
**HE**	0.833	0.744	-					
**AC**	0.844	0.740	0.877	-				
**AR**	0.696	0.654	0.675	0.768	-			
**PN**	0.735	0.718	0.819	0.747	0.602	-		
**GT**	0.700	0.644	0.813	0.755	0.585	0.821	-	
**OFC**	0.605	0.579	0.699	0.645	0.523	0.786	0.793	-

**Note:** HV—Health Values, HM—Health Motivation, HE—Healthy Eating Belief, AC—Awareness of Consequences, AR—Ascription of Responsibility, PN—Personal Norms, GT—Trust on Organic Food, OFC—Organic Food Consumption

### Structural model

To test the current model’s stability and assess its structural validity, this study utilized SmartPLS 3.0 to perform bootstrapping analysis on 5000 sub-samples at a significance level of 5%. This analysis calculated the coefficient of determination (*R*^*2*^) and effect sizes (*f*^*2*^) to evaluate the explanatory power of endogenous constructs and the magnitude of the model’s structural effects. Additionally, the blindfolding procedure was employed to compute predictive relevance (*Q*^*2*^) for assessing the model’s predictive capabilities.

The endogenous coefficients of determination (*R*^*2*^) in this study were all above the threshold value of 0.10 [83]. The research model explained 62.8% of the variance in healthy eating belief (*R*^*2*^ = 0.628), 68.4% of the variance in awareness of consequences (*R*^*2*^ = 0.684), 54.5% of the variance in ascription of responsibility (*R*^*2*^ = 0.545), 61.2% of the variance in personal norms (*R*^*2*^ = 0.612), and 64.9% of the variance in organic food consumption (*R*^*2*^ = 0.649). The predictive relevance of the model was tested by examining whether *Q*^*2*^ is greater than zero. As shown in Table 4, the *Q*^*2*^ values for healthy eating belief (0.475), awareness of consequences (0.595), ascription of responsibility (0.444), personal norms (0.523), and organic food consumption (0.542) were all positive and fairly substantial. Therefore, the measurement model exhibits satisfactory and substantial predictive capabilities.

**Table 4:** Hypothesis testing.

Hypothesis		Beta	CI-Min	CI-Max	*T Statistics*	*p Values*	*R* ^*2*^	*Q* ^*2*^	Decision
**H1**	**HV → HE**	0.561	0.553	0.064	8.744	0.000	0.628	0.475	Supported
**H2**	**HM → HE**	0.268	0.275	0.057	4.729	0.000			Supported
**H3**	**HE → AC**	0.827	0.825	0.030	27.454	0.000	0.684	0.595	Supported
**H4**	**AC → AR**	0.738	0.740	0.028	26.397	0.000	0.545	0.444	Supported
**H5**	**HE → PN**	0.532	0.526	0.069	7.712	0.000			Supported
**H6**	**AC → PN**	0.224	0.227	0.086	2.620	0.005	0.612	0.523	Supported
**H7**	**AR → PN**	0.075	0.076	0.056	1.341	0.090			Rejected
**H8**	**PN → OFC**	0.432	0.433	0.079	5.467	0.000	0.649	0.542	Supported
**Moderating effect of GT**									
**H9**	GT*PN → OFC	0.060	0.058	0.027	2.222	0.013			Moderate

**Note:** HV—Health Values, HM—Health Motivation, HE—Healthy Eating Belief, AC—Awareness of Consequences, AR—Ascription of Responsibility, PN—Personal Norms, GT—Green Trust, OFC—Organic Food Consumption

Effect size *f*^*2*^ is often used to determine the contribution of moderation to the explanation of the endogenous construct [84]. Based on Cohen’s [85] guidelines for interpreting *f*^*2*^, values greater than 0.02, 0.15, and 0.35 are considered small, medium, and large effect sizes, respectively. The results in Table 5 show that, except for the effect size between ascription of responsibility and personal norms (*f*^*2*^ = 0.007), all other *f*^*2*^ values exceed the minimum recommended value of 0.02. Notably, the effect of healthy eating beliefs on perception of consequences (*f*^*2*^ = 2.168) and perception of consequences on attribution of responsibility (*f*^*2*^ = 1.198) both had large effect size; health values on healthy eating beliefs (*f*^*2*^ = 0. 302), healthy eating beliefs on personal norms (*f*^*2*^ = 0.229), and personal norms on organic food consumption (*f*^*2*^ = 0.194) all had medium effect amount of effect.

**Table 5:** Effect size (*f*^*2*^*)*.

Variables	Healthy Eating Belief	Awareness of Consequences	Ascription of Responsibility	Personal Norms	Organic Food Consumption
**Health Values**	0.302				
**Health Motivation**	0.069				
**Healthy Eating Belief**		2.168		0.229	
**Awareness of Consequences**			1.198	0.031	
**Ascription of Responsibility**				0.007	
**Personal Norms**					0.194

### Hypothesis testing

The results of the path coefficients indicate that health values (β = 0.561, p<0.001, t>1.645) and health motivation (β = 0.268, p<0.001, t>1.645) have a positive and significant effect on healthy eating belief, supporting H1 and H2. Healthy eating belief (β = 0.827, p<0.001, t>1.645) has a positive and significant influence on awareness of consequences, supporting hypothesis 3. The study reveals that awareness of consequences (β = 0.738, p<0.001, t>1.645) positively and significantly affects ascription of responsibility, validating hypothesis 4. Healthy eating belief (β = 0.532, p<0.001, t>1.645) and awareness of consequences (β = 0.224, p<0.05, t>1.645) have a positive and significant impact on personal norms, supporting H5 and H6, consistent with previous hypotheses. However, there is no significant effect between ascription of responsibility (β = 0.075, p>0.05, t<1.645) and personal norms, leading to the rejection of hypothesis 7. Personal norms (β = 0.432, p<0.001, t>1.645) has a positive and significant influence on organic food consumption, supporting H8. Additionally, the moderation effect of green trust is confirmed, as green trust exhibits a positive and significant moderating effect (β = 0.060, p<0.05, t>1.645) in the relationship between personal norms and organic food consumption, supporting H9.

## Discussion

Owing to environmental degradation, resource scarcity, and food safety incidents, people are increasingly concerned about the role of food in their health and in protecting the environment [1, 5]. Organic food, in turn, is considered to play a dual role in protecting the environment and health of individuals [33]. Based on the value-belief-norm theory, this study explores the processes that lead to or reinforce the consumption of organic food due to consumers’ health concerns and trust on organic food, and examines the role of each factor at each stage. All hypotheses are supported, except for H7. Specifically, the following empirical results are presented and discussed.

First, research has demonstrated the positive effects of health values and health motivation on healthy eating beliefs. The relationship between value and belief in the VBN theory has been repeatedly validated in the extant literature [34]. The present study also yields the same result that individuals’ HV positively impact their HE. In addition, this study verifies the effect of HM on HE and obtains the same conclusion as McArthur et al. [86]. The findings suggest that individuals are more likely to believe that eating organic food is good for their health if they associate health with their well-being, and care for, and value their health, and are willing to learn about health and eat healthy food to maintain their health. The results also show that an individual’s HV has a greater impact on HE than on HM. This is consistent with the daily life. In other words, individuals who are aware of the importance of health are more likely to possess well-developed beliefs about healthy eating than if they have health knowledge and are motivated to eat healthy food.

Second, this study validated the causal chain of healthy eating beliefs, awareness of consequences, and ascription of responsibility. The relationship between belief, AC, and AR has also been mentioned in many studies based on the VBN theory [29, 52]. The results suggest that consumers’ knowledge of and trust in the health effects of organic food can lead them to focus on the good consequences of managing their health through a healthy diet. Their awareness of the consequences of their health behavior further leads them to make efforts and take responsibility for their health. We believe that HE and AC influence consumers to take responsibility for their health, possibly because of their egoistic mindset. Egoists are likelier to want to be healthy and dislike unhealthy lifestyles [53]. The starting point for HE and AC is the individuals’ concern for their health.

Third, the study found a predictive effect of healthy eating beliefs and awareness of consequences on personal norms, but a non-significant predictive effect of ascription of responsibility. We agree with the findings of Lind et al. [65] and Ünal et al. [31] regarding the relationship among HE, AC, and PN. The fact that H5 and H6 are supported suggests that the more the consumers believe that organic food can reduce cancer risk, improve gut health, and prevent health problems, and the more they understand the relationship between diet and health problems, the more likely they are to believe that food should be considered environmentally friendly and natural and that they should have a moral obligation to consume and promote organic food. This is because the more natural and organic the food, the more beneficial it will be for consumers’ health [55]. However, unexpectedly, no significant effect of AR on PN was detected in our findings, although the relationship between them was positive. This is in conflict with the findings of many scholars [28, 34]. Individuals generally activate personal norms when they know that they are responsible for their health [33]. There are two possible reasons due to which our results show no relationship between AR and PN. First, this could be a differential representation of the sample or a different characteristic of the youth in Bangladesh. Second, the measured items for PN also included questions about environmental protection (related to altruism), whereas AR is entirely about the responsibility that individuals take for themselves and it may be more related to egoism, which may lead to a non-significant relationship between AR and PN. Based on these results and perspectives, it is possible to conclude that individuals generate norms about self-health constraints, possibly due to the presence of egoism [53, 61].

Fourth, personal norms directly predict organic food consumption and that trust on organic food positively moderates the relationship between them. This result reconfirms that personal norms are a direct predictor of behavior [23]. This suggests that individuals who feel obliged to consume and promote organic food or who consider the environmental and sustainability aspects behind their food choices, are likely to identify with organic food purchasing behavior and choose organic food in their daily lives. Finally, Green trust has a positive and significant moderating effect on the relationship between personal norms and organic food consumption. Green trust provides customers with reasons to choose organic food, attracting their attention. Trust in green products helps organic food to stand out from similar products, and when organic food meets consumers’ personal expectations, it will drive consumers’ consumption of organic food. The results in this study again validate this conclusion, those consumers who perceive organic food as reliable and trustworthy, and who consider organic food to be environmentally protective, catalyze the process of moving from internal psychological activities to external behavioral manifestations when their moral obligations coincide with the consumption of organic food, thus implying that they are more likely to purchase organic food.

## Implications

### Theoretical Implications

This study is based on VBN theory combined with the health belief model and norm activation theory to construct a comprehensive conceptual model that considers the complex relationships between health values, health motivations, healthy eating beliefs, consequence awareness, responsibility attribution, personal norms, and organic food consumption among young Bangladeshi consumers. This study not only validated the causal chain between values, beliefs, and norms in VBN theory but also explored the influence of health-related values, motivations and beliefs on personal norms and organic food consumption. In addition, this study found a moderating effect of consumer trust in organic food on the relationship between personal norms and organic food consumption. These results enrich the understanding of the relationship between personal norms and organic food consumption in the existing literature and demonstrate the complementarity between different theoretical frameworks. In conclusion, this paper provides an in-depth study of the mechanisms influencing consumers’ organic food consumption behavior, thus enriching the application of the VBN theory to the organic food sector and providing a reference for localized research in Bangladesh.

Furthermore, theoretically, this study shows that personal norms are formed by a combination of factors, including values, beliefs, motivations, and awareness. These factors can explain the formation process of personal norms at different levels, such as values and beliefs at the cognitive level, motivation at the affective level, and norms at the moral level. In addition, this study examines the influence of personal norms on behavior from different perspectives, such as egoism (concern for one’s own health), altruism (concern for environmental protection), and trust (trustworthiness of organic food). The interaction and influence of these factors can provide new perspectives and ideas for the development and extension of subsequent research on personal norms.

### Practical implications

This study was conducted in the specific cultural and social context of Bangladesh, a developing country with values, beliefs, and behavioral patterns different from those of Western countries. Bangladesh is a largely agricultural country, rich in natural resources and biodiversity, but also faces challenges such as environmental degradation, food safety and malnutrition, and organic food is still in its infancy in Bangladesh, lacking policy support, certification mechanisms, market channels, and consumer awareness [87]. Although households with older people and children are encouraged to purchase organic food as a regular program in Bangladesh [88], there is still a need for more research on how to guide people to encourage consumption of organic food in households. This study focuses on the personal health management aspects of organic food consumption to investigate the causes of consumer behavior. At the macro level, this research can inform public sector policies and actions to guide consumption, for example, by promoting health management through official media channels; sharing knowledge about organic food, and promoting the natural, healthy, and environment friendly properties of organic food. Simultaneously, the public sector could also consider developing subsidies related to organic food, either for producers and enterprises to subsidize production costs or for consumers to subsidize purchase costs. At the micro level, our study provides guidance for organic food producers and businesses to develop proper marketing strategies. When marketing organic food, producers and businesses should consider consumers’ self-interest, and in addition to the environmental perspective, should increase their efforts to promote the health-related benefits of organic food, such as cancer prevention, gut health improvement, and diabetes management. Furthermore, the quality of organic food production should be strictly controlled by producers, and the certification of organic food should be provided by the public sector. This will increase consumer confidence in organic food and encourage them to buy it.

## Conclusions

Organic food has attracted much attention from scholars because of its dual health and environmental properties [89]. However, there are few studies on organic food consumption in Bangladesh from a health-management perspective. In this study, we used PLS-SEM to analyze a range of factors and processes that can lead to organic food consumption among the youth in Bangladesh. Our empirical results show that the most direct influence on organic food consumption is personal norms and that trust on organic food positively moderates the relationship between them. At the same time, it has been shown that health values and health motivation can positively influence healthy eating beliefs, and that healthy eating beliefs, awareness of consequences, and ascription of responsibility form a complete causal chain. However, we only found a predictive effect of healthy eating beliefs and awareness of consequences on personal norms but not a significant effect of ascription of responsibility. This study has implications for the promotion of organic food to consumers.

There are still many aspects that could be improved upon in regards to this study. First, our sample comprises young Bangladeshi people, which only covers a part of the age range, and this may not provide an accurate picture of whether the behavior of consuming organic food is different for different age groups. Therefore, in future studies, the sample should be broadened to cover a wider range of ages to obtain more representative results. Second, we only analyzed the two-way interaction of factors ranging from health values and health motivation to healthy eating beliefs, awareness of consequences, attribution of responsibility, and personal norms, which form a relatively complete causal chain, but we did not consider the mediating role of some of these factors. Therefore, future research should further test the mediating effects of these factors. Third, we collected the demographic characteristics of the respondents, but we did not conduct subgroup comparison analyses to test whether different results were presented across different population characteristics. Therefore, we suggest that future scholars analyze the differential performance of different population characteristics and draw new conclusions.

## Supporting information

S1 Dataset (CSV)

S1 Table Survey questionnaire. (DOCX)

S2 Table Discriminant validity. (DOCX)
