# Supplementary material for: Modelling the mass consumption potential of organic food: Evidence from an emerging economy
Source: PLoS One. 2023 Sep 1;18(9):e0291089. doi: 10.1371/journal.pone.0291089 (PMC10473519; doi:10.1371/journal.pone.0291089)
Supplement: S1 Table — (DOCX) [file pone.0291089.s002.docx]

**Supporting Material S1.** Survey Questionnaire

| Code | | Items | |
| --- | --- | --- | --- |
| HV1 | | If I don’t have my health, I don’t have anything | |
| HV2 | | There is nothing I care more about than my health | |
| HV3 | | Good health is most important for happy life | |
| HV4 | | Nothing is more important than good health | |
| HM1 | | I usually value my health. | |
| HM2 | | I take every action to eat healthy food | |
| HM3 | | I have good knowledge to prevent health issues | |
| HM4 | | I am confident that I can maintain my health | |
| HM5 | | Prevention is the best strategy to keep me healthy | |
| HE1 | I believe eating plenty of vegetables, fruits, and whole grains good for my health | | |
| HE2 | I believe eating organic food can reduce cancer risk | | |
| HE3 | I believe eating organic food can improve gut health | | |
| EV4 | I believe eating organic food can improve diabetes management | | |
| EV5 | I believe eating organic food can prevent many health issues | | |
| AC1 | Personal health management can improve the individual’s quality of life. | | |
| AC2 | Adaptation of healthy eating practicebs can reduce the negative consequences towards personal health. | | |
| AC3 | Health issues can be curtailed with the management of personal diet | | |
| AC4 | Minor health concerns may lead to grave health issues | | |
| AC5 | Overall, eating organic food can improve personal health. | | |
| AR1 | I think taking responsibility for personal health is important. | | |
| AR2 | I feel that taking responsibility for personal healthcare can help to promote a healthy society. | | |
| AR3 | Taking personal responsibility for healthcare to promote personal well-being. | | |
| AR4 | Everyone must take responsibility for personal health. | | |
| AR5 | I feel responsible for personal healthcare. | | |
| PN1 | I feel morally obliged to consume organic food | | |
| PN2 | People like me should do everything they can to eat organic food | | |
| PN3 | I feel obliged to bear the environment and nature in mind in my food consumption behaviours | | |
| PN4 | I feel morally obliged to consume organic products, regardless of what others do | | |
| PN5 | I feel personally obliged to promote organic food and organic food production methods | | |
| GT1 | | | Organic foods are generally reliable |
| GT2 | | | Organic food’s environmental performance is generally dependable |
| GT3 | | | Organic foods are generally trustworthy |
| GT4 | | | Organic foods meets my expectations |
| GT5 | | | Organic production method’s keep promises for environmental protection |
| OFC1 | I frequently consume organic food | | |
| OFC2 | I intentionally purchase organic food grown in an energy efficient environment | | |
| OFC3 | I frequently pay premium price for organic food | | |
| OFC4 | I set a positive example by consuming organic food for my community | | |
| OFC5 | I encourage my friends and relatives to consume organic food | | |

**Note:** HV - Health Values, HM - Health Motivation, HE - Healthy Eating Belief, AC - Awareness of Consequences, AR - Ascription of Responsibility, PN - Personal Norms, GT - Green Trust, OFC - Organic Food Consumption
