# Supplementary material for: Modelling the mass consumption potential of organic food: Evidence from an emerging economy
Source: PLoS One. 2023 Sep 1;18(9):e0291089. doi: 10.1371/journal.pone.0291089 (PMC10473519; doi:10.1371/journal.pone.0291089)
Supplement: S2 Table — (DOCX) [file pone.0291089.s003.docx]

**Supporting Material S2.** Discriminant Validity

|  | HV | HM | HE | AC | AR | PN | GT | OFC |
| --- | --- | --- | --- | --- | --- | --- | --- | --- |
| ﻿HV1 | 0.939 | 0.778 | 0.752 | 0.785 | 0.672 | 0.662 | 0.640 | 0.529 |
| HV2 | 0.931 | 0.719 | 0.687 | 0.734 | 0.585 | 0.638 | 0.616 | 0.541 |
| HV3 | 0.954 | 0.783 | 0.763 | 0.794 | 0.633 | 0.707 | 0.661 | 0.560 |
| HV4 | 0.952 | 0.746 | 0.726 | 0.754 | 0.626 | 0.661 | 0.621 | 0.557 |
| HM1 | 0.778 | 0.779 | 0.757 | 0.801 | 0.697 | 0.636 | 0.638 | 0.573 |
| HM2 | 0.487 | 0.775 | 0.366 | 0.408 | 0.356 | 0.444 | 0.346 | 0.337 |
| HM3 | 0.575 | 0.838 | 0.449 | 0.477 | 0.433 | 0.538 | 0.456 | 0.417 |
| HM4 | 0.529 | 0.812 | 0.401 | 0.415 | 0.387 | 0.474 | 0.410 | 0.362 |
| HM5 | 0.739 | 0.875 | 0.716 | 0.723 | 0.621 | 0.645 | 0.608 | 0.512 |
| HE1 | 0.499 | 0.576 | 0.532 | 0.429 | 0.315 | 0.461 | 0.468 | 0.309 |
| HE2 | 0.716 | 0.641 | 0.941 | 0.759 | 0.593 | 0.695 | 0.693 | 0.595 |
| HE3 | 0.721 | 0.638 | 0.960 | 0.796 | 0.621 | 0.721 | 0.727 | 0.636 |
| HE4 | 0.721 | 0.655 | 0.937 | 0.779 | 0.622 | 0.738 | 0.692 | 0.654 |
| HE5 | 0.718 | 0.653 | 0.938 | 0.797 | 0.579 | 0.702 | 0.712 | 0.632 |
| AC1 | 0.756 | 0.683 | 0.749 | 0.916 | 0.671 | 0.622 | 0.632 | 0.508 |
| AC2 | 0.781 | 0.713 | 0.786 | 0.949 | 0.706 | 0.687 | 0.692 | 0.587 |
| AC3 | 0.789 | 0.745 | 0.769 | 0.952 | 0.705 | 0.686 | 0.684 | 0.580 |
| AC4 | 0.757 | 0.706 | 0.774 | 0.939 | 0.698 | 0.687 | 0.691 | 0.594 |
| AC5 | 0.722 | 0.670 | 0.793 | 0.925 | 0.675 | 0.686 | 0.704 | 0.632 |
| AR1 | 0.617 | 0.589 | 0.574 | 0.686 | 0.884 | 0.525 | 0.492 | 0.465 |
| AR2 | 0.590 | 0.623 | 0.587 | 0.673 | 0.930 | 0.550 | 0.524 | 0.455 |
| AR3 | 0.632 | 0.636 | 0.590 | 0.693 | 0.928 | 0.560 | 0.548 | 0.471 |
| AR4 | 0.649 | 0.628 | 0.634 | 0.721 | 0.926 | 0.555 | 0.562 | 0.479 |
| AR5 | 0.541 | 0.513 | 0.499 | 0.576 | 0.890 | 0.434 | 0.417 | 0.402 |
| PN1 | 0.662 | 0.634 | 0.706 | 0.676 | 0.550 | 0.917 | 0.752 | 0.728 |
| PN2 | 0.665 | 0.670 | 0.720 | 0.695 | 0.593 | 0.934 | 0.765 | 0.725 |
| PN3 | 0.639 | 0.652 | 0.701 | 0.654 | 0.532 | 0.930 | 0.705 | 0.643 |
| PN4 | 0.647 | 0.647 | 0.698 | 0.646 | 0.512 | 0.935 | 0.711 | 0.674 |
| PN5 | 0.674 | 0.642 | 0.732 | 0.673 | 0.503 | 0.933 | 0.737 | 0.731 |
| GT1 | 0.657 | 0.611 | 0.733 | 0.705 | 0.575 | 0.755 | 0.932 | 0.706 |
| GT2 | 0.638 | 0.610 | 0.747 | 0.720 | 0.549 | 0.745 | 0.941 | 0.698 |
| GT3 | 0.588 | 0.585 | 0.655 | 0.611 | 0.494 | 0.710 | 0.930 | 0.672 |
| GT4 | 0.620 | 0.607 | 0.670 | 0.629 | 0.479 | 0.745 | 0.927 | 0.752 |
| GT5 | 0.624 | 0.572 | 0.725 | 0.719 | 0.518 | 0.719 | 0.921 | 0.705 |
| OFC1 | 0.537 | 0.521 | 0.600 | 0.553 | 0.435 | 0.686 | 0.681 | 0.897 |
| OFC2 | 0.480 | 0.493 | 0.567 | 0.549 | 0.445 | 0.655 | 0.685 | 0.920 |
| OFC3 | 0.542 | 0.534 | 0.614 | 0.578 | 0.454 | 0.704 | 0.704 | 0.931 |
| OFC4 | 0.522 | 0.514 | 0.571 | 0.548 | 0.447 | 0.697 | 0.700 | 0.931 |
| OFC5 | 0.580 | 0.560 | 0.679 | 0.624 | 0.518 | 0.725 | 0.728 | 0.921 |

**Note:** HV - Health Values, HM - Health Motivation, HE - Healthy Eating Belief, AC - Awareness of Consequences, AR - Ascription of Responsibility, PN - Personal Norms, GT - Green Trust, OFC - Organic Food Consumption
